# Genome-wide mapping of nucleosome positions in *Saccharomyces cerevisiae* in response to different nitrogen conditions

**DOI:** 10.1038/srep33970

**Published:** 2016-09-23

**Authors:** Peng Zhang, Guocheng Du, Huijun Zou, Guangfa Xie, Jian Chen, Zhongping Shi, Jingwen Zhou

**Affiliations:** 1 Key Laboratory of Industrial Biotechnology, Ministry of Education and School of Biotechnology, Jiangnan University, 1800 Lihu Road, Wuxi, Jiangsu 214122, China; 2 Zhejiang Guyuelongshan Shaoxing Wine Company, 13 Yangjiang Road, Shaoxing, Zhejiang, China

## Abstract

Well-organized chromatin is involved in a number of various transcriptional regulation and gene expression. We used genome-wide mapping of nucleosomes in response to different nitrogen conditions to determine both nucleosome profiles and gene expression events in *Saccharomyces cerevisiae*. Nitrogen conditions influence general nucleosome profiles and the expression of nitrogen catabolite repression (NCR) sensitive genes. The nucleosome occupancy of TATA-containing genes was higher compared to TATA-less genes. TATA-less genes in high or low nucleosome occupancy, showed a significant change in gene coding regions when shifting cells from glutamine to proline as the sole nitrogen resource. Furthermore, a correlation between the expression of nucleosome occupancy induced NCR sensitive genes or TATA containing genes in NCR sensitive genes, and nucleosome prediction were found when cells were cultured in proline or shifting from glutamine to proline as the sole nitrogen source compared to glutamine. These results also showed that variation of nucleosome occupancy accompany with chromatin-dependent transcription factor could influence the expression of a series of genes involved in the specific regulation of nitrogen utilization.

Nucleosomes form the basic repeating unit of eukaryotic chromatin, consisting of ~147 bp DNA wrapped around a histone octamer core^1^,^2^,^3^. Dynamic changes of genome-wide maps of nucleosome positions reflect a set of molecular processes, including DNA replication, gene expression and transcription regulation^4^,^5^. Upon changes in response to different environmental conditions, >20% genes switch their promoter states^6^. DNA-binding transcription factors and chromatin-remodeling enzymes are primarily responsible for the nucleosome architecture^6^. Meanwhile, different nucleosome profiles could be observed under the same culture conditions while cells’ growth conditions are distinct^7^. Precise nucleosome positions on the genome inhibit the binding of any other protein, such as a transcriptional activator or the general transcriptional machinery, to the target DNA region. The change of nucleosome positions is crucial for understanding the mechanism underlying the regulation of gene expression^8^.

In response to different nutritional conditions or perturbation of the environment, yeast cells tend to fine tune their transcriptional, translational and metabolic processes in order to maximize survival ability^9^. For different nitrogen sources, including preferred nitrogen sources glutamate and glutamine or non-preferred nitrogen sources, such as proline, *Saccharomyces cerevisiae* is able to use the nitrogen source that enables best growth by a mechanism called nitrogen catabolite repression (NCR)^10^,^11^,^12^. The NCR genes are regulated primarily by an interplay of the four GATA family transcription factors transcriptional activators Gln3 and Gat1 as well as repressors Dal80 and Gzf3^13^,^14^. More than 392 genes are involved in the response to changes of nitrogen source type and quality and about 90 genes are regulated directly by the four GATA regulators^9^,^14^,^15^. These NCR-sensitive genes are involved in some metabolic processes, including amino acid and allantoin metabolism as well as transport and transcription regulation.

Recent advances in high-throughput sequencing technology have dramatically expanded our understanding of genome-wide nucleosome organization^6^,^16^,^17^. The nucleosome core can be isolated as a particle containing 147 bp DNA by the digestion of chromatin with micrococcal nuclease (MNase)^18^,^19^. MNase-seq is considered the standard procedure for nucleosome profile prediction^6^,^20^. Nucleosomes overlapping the transcription start site (TSS) of gene-coding regions are defined as +1 nucleosomes, while a nucleosome immediately before a +1 nucleosome is defined as a −1 nucleosome, the so called +1 and −1 nucleosomes flank a considerably larger opening termed the nucleosome-free region (NFR)^6^. Evidence is presented that the NFRs at TSS is essential for successful transcription initiation^21^,^22^. A 3′ NFR present in >95% of all genes might be important in transcription termination;^23^ however, the dynamic change of nucleosome positions in response to different nitrogen conditions has not been examined on a genome-wide scale in *S. cerevisiae*. To map the location of individual nucleosomes on a genomic scale in response to different nitrogen conditions, we used a MNase-seq method to sequence the ends of nucleosome-associated DNA. This study focused firstly on the regions around TSSs and stop codon sites, which are well-defined nucleosome distributions^23^,^24^. Furthermore, the nucleosome profiles of the TATA-containing and TATA-less promoters were analyzed to determine the correlation between general gene distribution and nucleosome profiles. Finally, the expression of NCR-sensitive genes was calculated to investigate their relationship under different nitrogen conditions.

## Results

### Nucleosome profiles of the 5′ and 3′ ends of all genes under different nitrogen conditions

**Figure 1:**
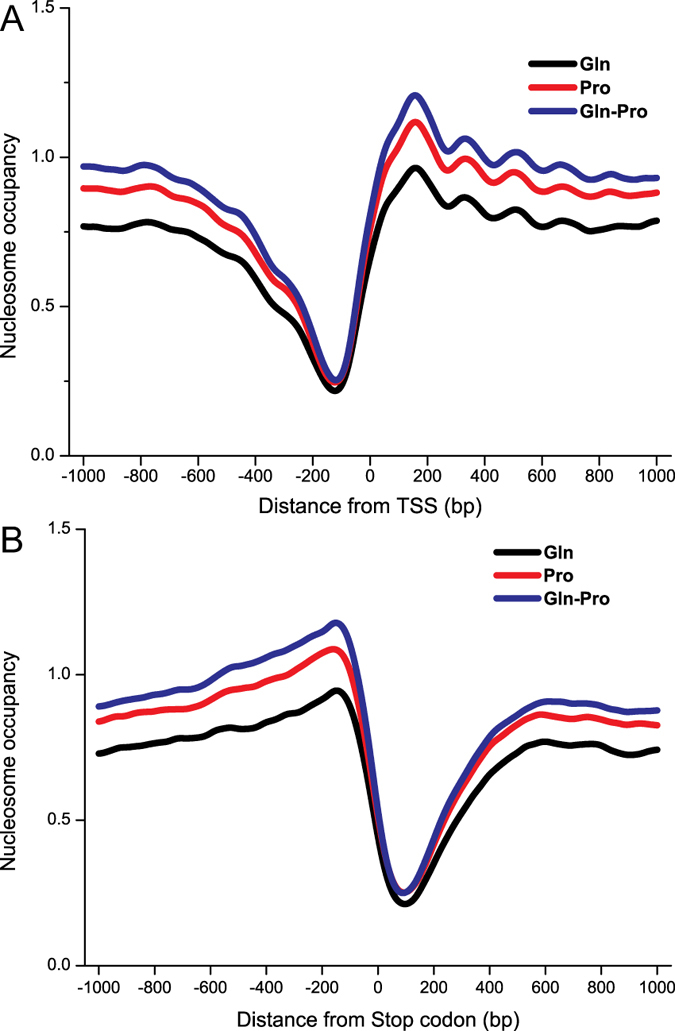
Patterns of nucleosome occupancy of *S. cerevisiae* under different nitrogen conditions. (**A**) Nucleosome occupancy profiles of all genes in the yeast genome from −1000 bp to +1000 bp with respect to the TSS. (**B**) Nucleosome occupancy profiles of all genes in the yeast genome from −1000 bp to +1000 bp with respect to the stop codon.

Knowledge of the detailed remodeling of nucleosome positioning across genomes and the mechanism underlying nucleosome profile changes is crucial for understanding gene regulation and expression. The nucleosome profiles of the 5′ and 3′ ends of a gene-coding region have been considered as a marker for gene transcription initiation and termination. Here, the whole genome data of nucleosome occupancy profiles were exhibited at the 5′ and 3′ ends of all genes (Fig. 1A,B). There is a notable NFR immediately upstream of the TSS and downstream of the stop codon; the high nucleosome occupancy region is immediately downstream of the TSS and upstream of the stop codon. Therefore, the nucleosome occupancy score differed under different nitrogen conditions: Gln, low; Pro, medium; and Gln-Pro, high.

### Nucleosome profiles of TATA-containing and TATA-less genes under different nitrogen conditions

**Figure 2:**
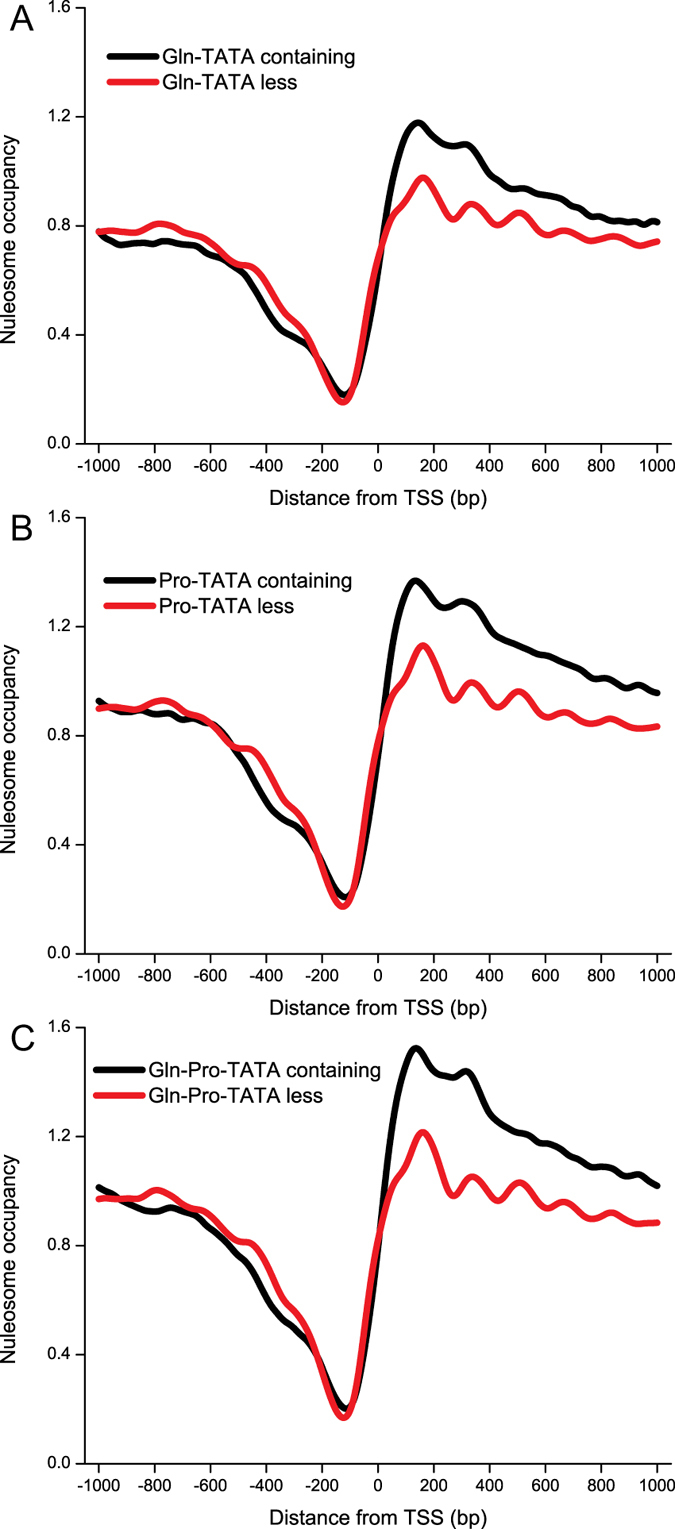
Nucleosome occupancy of TATA-containing and TATA-less genes in response to different nitrogen conditions. Nucleosome occupancy for TATA-containing (973) and TATA-less (4382) promoters, aligned with respect to the TSS. (**A**) Nucleosome profiles of *S. cerevisiae* in M.Gln. (**B**) Nucleosome profiles of *S. cerevisiae* in M.Pro. (**C**) Nucleosome profiles of *S. cerevisiae* for M.Gln shift to M.Pro for 2 h.

Approximately 20% of yeast genes contain a TATA box. Based upon the presence or absence of a TATA box in the promoter region, the genes can be classified as TATA-containing or TATA-less genes^25^. Plots of the nucleosome profiles on their promoter revealed the differences of nucleosome occupancy on the TATA-containing and TATA-less genes under different nitrogen conditions (Fig. 2). There were no significant differences of the general nucleosome occupancy profiles between TATA-containing and TATA-less genes, and the general genes under different nitrogen conditions (Gln, low; Pro, medium; and Gln-Pro, high.). TATA-containing genes downstream of TSS had a higher nucleosome occupancy score compared to TATA-less genes; however, there was no significant difference upstream of TSS.

### Analysis of nucleosome profiles and general gene distribution

**Figure 3:**
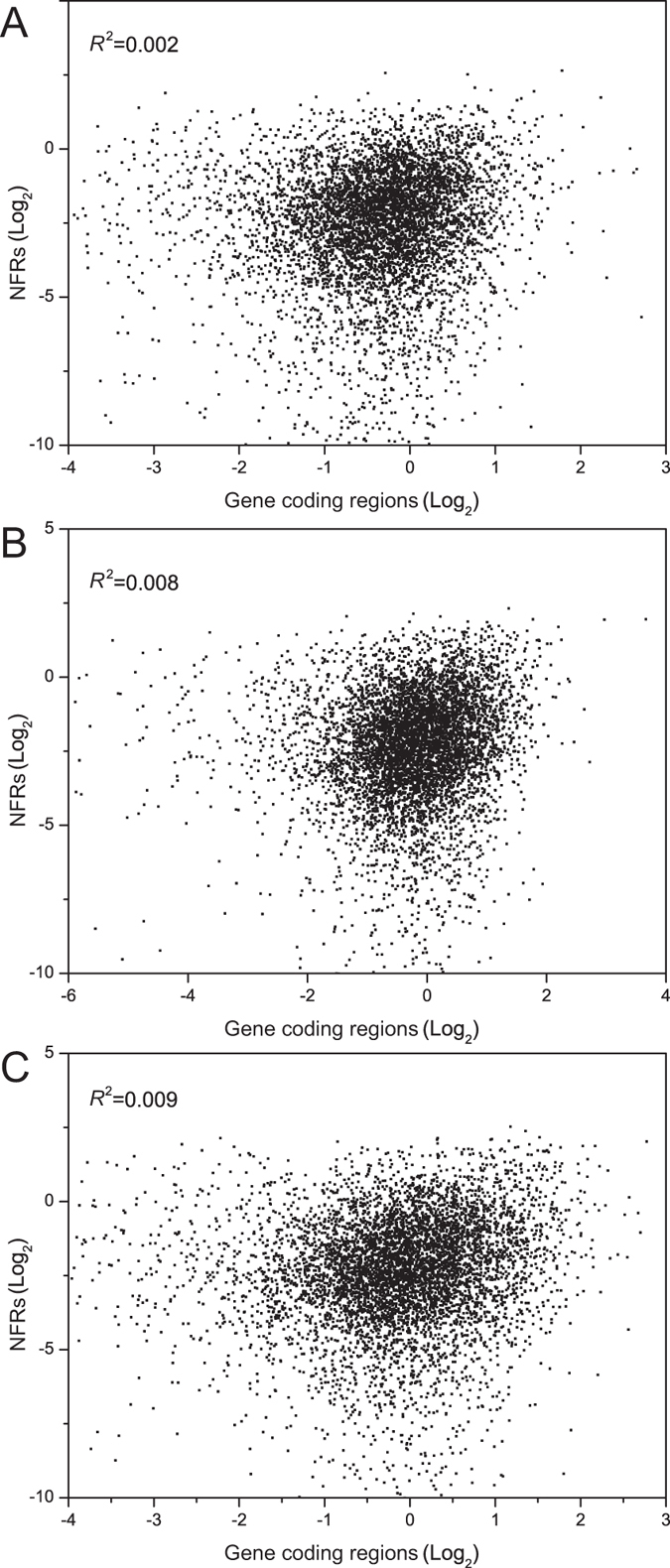
Correlation between nucleosome occupancy at NFRs and gene coding regions. There was no correlation between nucleosome occupancy at NFRs and gene coding regions. The nucleosome occupancy at NFRs were plotted against gene coding regions under three nitrogen conditions, and no correlation was observed with *R*^2^ < 0.009. (**A**) Correlation between nucleosome occupancy at NFRs and gene coding regions in M.Gln medium. (**B**) Correlation between nucleosome occupancy at NFRs and gene coding regions in M.Pro medium. (**C**) Correlation between nucleosome occupancy at NFRs and gene coding regions in response to the shift from glutamine to proline for 2 h.

**Figure 4:**
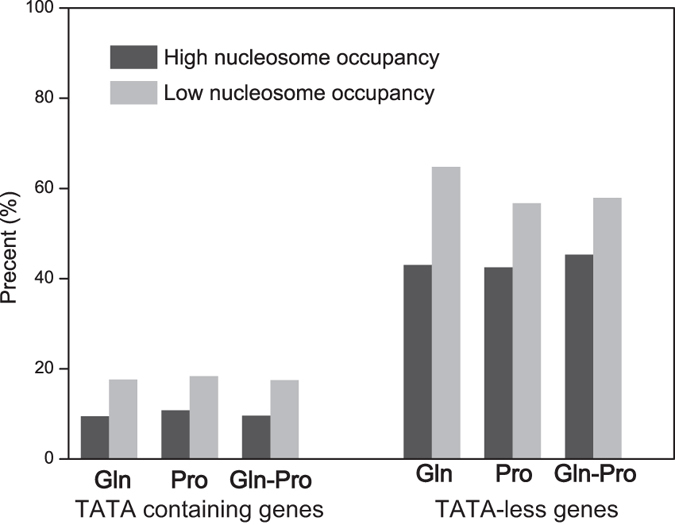
Percentage of TATA-containing and TATA-less genes with high or low nucleosome occupancy. Distribution of TATA-containing and TATA-less genes with high or low nucleosome occupancy. Gln, Pro and Gln-Pro represent the culture conditions in M.Gln, M.Pro and in response to the shift from M.Gln to M.Pro for 2 h, respectively.

**Figure 5:**
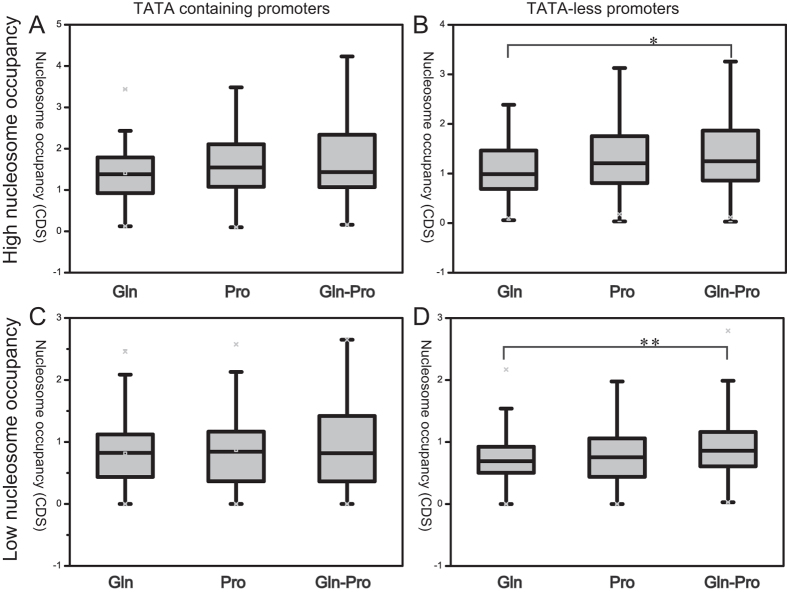
Correlation between nucleosome occupancy at NFRs and gene coding regions for TATA-containing and TATA-less promoters. (**A**) TATA-containing; (**B**) TATA-less promoters with high nucleosome occupancy; (**C**) TATA-containing; (**D**) TATA-less promoters with low nucleosome occupancy. Gln, Pro and Gln-Pro represent the culture conditions in M.Gln, M.Pro and in response to the shift from M.Gln to M.Pro for 2 h, respectively. *P*-values were calculated using the Wilcoxon rank sum test, *P* < 0.05 (*), < 0.01 (**) and <0.001 (***).

The nucleosome occupancy score of NFRs and genes coding regions were calculated to investigate their relationships under different nitrogen conditions. There was no significant relationship (*R*^2^ < 0.009) between the nucleosome occupancy amount of gene coding regions and NFRs (Fig. 3). Thus, nucleosome occupancy at NFRs and gene types were examined further. Nucleosome occupancy score in the 10% most strongly positioned nucleosome (High nucleosome occupancy) and the 10% most weakly positioned nucleosomes (low nucleosome occupancy) over the NFRs were used for correlation analysis of nucleosome occupancy at NFRs and the distribution of TATA-containing and TATA-less genes. Figure 4 shows the NFRs of TATA-less genes are frequently covered with high and low nucleosome occupancy. We compared the correlation between normalized nucleosome occupancy scores of gene coding regions and NFRs of TATA-containing and TATA-less genes with high and low nucleosome occupancy (Fig. 5). The nucleosome occupancy score at the coding regions of TATA-containing genes showed no significant change in response to different nitrogen conditions. However, the nucleosome occupancy at coding regions of TATA-less genes while shift from M.Gln to M.Pro was changed significantly with high and low nucleosome occupancy at NFRs compared to M.Gln.

### Analysis of the nucleosome occupancy and gene expression of nitrogen metabolic genes

**Figure 6:**
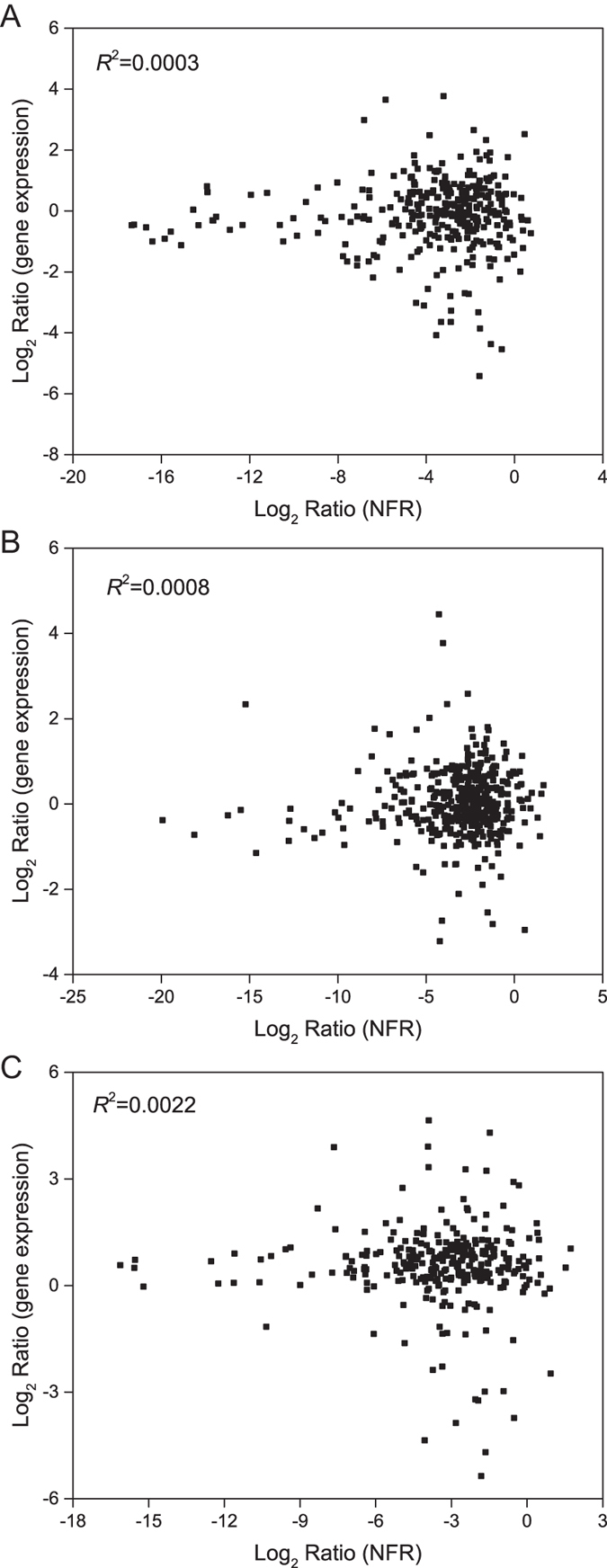
Correlation between nucleosome occupancy at NFRs and the expression of nitrogen metabolic genes. Correlation between the nucleosome occupancy at NFRs and the expression of 506 nitrogen metabolic genes were analyzed under different nitrogen conditions. (**A**) Correlation between the nucleosome occupancy and the expression of nitrogen metabolic genes in M.Gln medium; (**B**) Correlation between the nucleosome occupancy and the expression of nitrogen metabolic genes in M.Pro medium; (**C**) Correlation between the nucleosome occupancy and the expression of nitrogen metabolic genes in M.Gln-Pro medium.

In order to understand the correlation between nucleosome occupancy and gene expression under different nitrogen conditions, we compared the gene expression of 506 nitrogen metabolic genes that had reported previously^15^ and the nucleosome occupancy at NFR. As shown in Fig. 6, there are no significant correlation (*R*^2^ < 0.0022) between the nucleosome occupancy and nitrogen metabolic gene expression under different nitrogen conditions, although the correlation in M.Gln-Pro medium (Fig. 6C) showed a little higher than that in M.Gln medium (Fig. 6A) and in M.Pro (Fig. 6B).

### Analysis of the nucleosome occupancy and gene expression of NCR-sensitive genes

**Figure 7:**
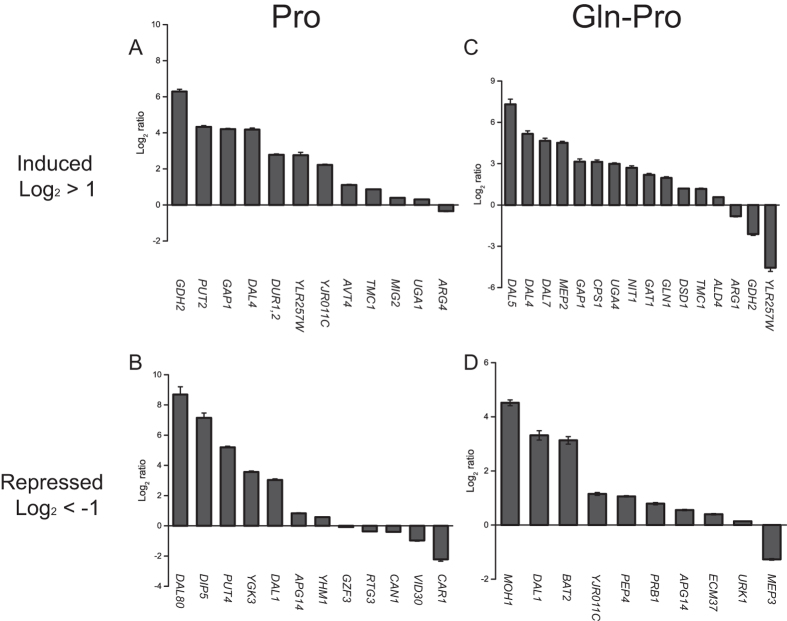
Comparison of nucleosome profile prediction and NCR-sensitive genes. The dark grey column represents the strength of gene expression detected by qRT-PCR. Each gene was examined for three times. (**A**) The expression of nucleosome profiles activated genes in M.Pro medium; (**B**) The expression of nucleosome profiles repressed genes in M.Pro medium; (**C**) The expression of nucleosome profiles activated genes in M.Gln-Pro medium; (**D**) The expression of nucleosome profiles repressed genes in M.Gln-Pro medium.

In order to further examine the nucleosome profile prediction on NCR sensitive genes expression, more than 80 well-characterized NCR-sensitive genes were used to demonstrate their nucleosome occupancy and gene expression in response to different nitrogen conditions. Compared to M.Gln, the remodeling profiles at NFRs that were activated or repressed at least 2.0-fold (Log_2_^ratio^ > 1 or Log_2_^ratio^ < −1) and *q*-value < 1.0E-30 under nitrogen source change were analyzed (Supplementary Table S4). Based upon the nucleosome profiles prediction results, 24 and 26 genes were identified in M.Pro and M.Gln-Pro media, respectively by qRT-PCR. As shown in Fig. 7, when the nucleosome profiles were activated, most of these target genes were up-regulated in M.Pro (Fig. 7A) and M.Gln-Pro (Fig. 7C) media. While the nucleosome profiles were repressed, the gene expression of target genes showed no significant correlation related to nucleosome profiles change under nitrogen condition change, especially in M.Gln-Pro medium (Fig. 7B,D). Furthermore, compared to M.Gln, the nucleosome profiles of the TATA containing genes of NCR sensitive genes at NFRs were preferred to be activated in M.Pro and M.Gln-Pro; the nucleosome profiles of TATA-less genes of NCR sensitive genes at NFRs were preferred to be repressed (Supplementary Table S4). This phenomenon is especially obvious in M.Gln-Pro. When *S cerevisiae* was shifted from preferred nitrogen conditions to non-preferred nitrogen conditions, most TATA-containing genes of NCR sensitive genes were up-regulated according to nucleosome profile activation, while most TATA-less genes of NCR sensitive were also up-regulated even their nucleosome profiles were repressed.

## Discussion

The development of high-throughput sequencing methods has provided global nucleosome positions across the genome of various eukaryotic organisms and environmental conditions^6^,^26^. Much experimental work has focused on determining the relationship between genome-wide nucleosome positioning and the regulation of gene expression and how these changes of nucleosome positioning influence gene expression^27^. In this study, dynamic remodeling of nucleosomes in the *S. cerevisiae* S288C genome was mapped under different nitrogen conditions using a combination of micrococcal nuclease digestion, mononucleosome DNA isolation and the Illumina high-throughput sequencing technologies. Analysis of the response of nucleosome profiles to different nitrogen conditions showed the nucleosome profiles associated with different gene types and the expression of NCR-sensitive genes, especially for those TATA containing genes in NCR sensitive genes.

Dynamic remodeling of individual nucleosomes across the yeast genome changed in response to transcriptional perturbation^27^. The well-positioned nucleosome profiles around the TSS and 3′ end stop codon site were changed under different nitrogen conditions (Fig. 1). However, under each condition, TATA-containing genes showed higher nucleosome occupancy in +1 nucleosome regions compared to TATA-less genes (Fig. 2). Since TATA-containing genes associated with response to stress are highly regulated by nucleosomal and TATA-binding protein-targeted mechanisms^28^, it appears the TATA-containing genes were also affected easily under these nitrogen conditions. Meanwhile, compared to M.Gln, the nucleosome profile of TATA-containing genes was also easily changed while shifting from glutamine to proline for 2 h (Fig. 2A,C). It means that TATA-containing genes are likely to undergo nucleosome profiles changes^29^. By comparing the nucleosome occupancy score at gene coding regions of TATA-containing and TATA-less genes with high or low nucleosome occupancy, TATA-less genes displayed a high average nucleosome occupancy in coding regions in M.Gln-Pro medium (Fig. 5). This phenomenon is also similar with nitrogen depletion in fission yeast^30^.

Glutamine and proline were preferred and non-preferred nitrogen sources, respectively, in response to NCR regulation in *S. cerevisiae* S288C^13^. Upon comparing glutamine and proline, shifting from a preferred nitrogen source (glutamine) to a non-preferred nitrogen source (proline), the expression of a set of genes involving amino acid and nucleotide metabolism, protein biosynthesis and degradation, stress response, molecule transport and unknown biological processes are changed^14^. The NCR-sensitive genes are involved in a set of regulatory mechanisms, including general amino acid control (GAAC), and the unfolded protein response as well as the SPS (Ssy1-Ptr3-Ssy5)-sensing system^12^,^15^. As shown in Fig. 6, the NCR-sensitive genes *GAP1* and *CAN1* were regulated by both NCR and GAAC, whereas *MEP2, MEP3, DAL5, UGA4, PUT4* and *DIP5* were regulated by NCR only^12^. The different regulatory mechanism might influence the correlation between gene expression and nucleosome prediction.

A shift from a preferred to a non-preferred nitrogen source results in a number of transcriptional changes^9^. A correlation between nucleosome profiles prediction and expression of NCR-sensitive genes was shown by comparing the NCR-sensitive gene expression with nucleosome occupancy changes and qRT-PCR (Fig. 7). Most of the nucleosome occupancy induced genes were associated with their qRT-PCR result, while the nucleosome occupancy repressed genes showed no correlation. Interestingly, shifting from glutamine to proline, most of the nucleosome occupancy induced NCR sensitive genes were TATA-containing genes, while most of the repressed genes were TATA-less genes (Supplement Table 4), Traditionally, a genome-wide remodeling of nucleosome profiles is related to two main types of regulation, ATP-dependent slip or eviction and post-modification of histone tails^31^. It is shown recently that the precise replacement of individual nucleosomes at promoters mechanistically regulates transcription also by modulating access of *trans*-acting factors to specific sites^27^. Hence, the expression of genes influenced by both nucleosome profile changes of transcriptional factor regulation is more easily distinguish from nucleosome prediction. In non-preferred nitrogen conditions, such as proline or shifting from glutamine to proline, two GATA transcriptional activators, Gln3p and Gat1p activate the NCR-sensitive genes for the utilization of non-preferred nitrogen source and cell growth^9^,^12^. Since the NCR-related proximal promoter motif, *GAT1*/*GLN3*/*DAL80* occurs preferentially in TATA-less promoters and show a strong preference for transcriptional factor binding^32^, the GATA factor binding might disturb the nucleosome prediction of the nucleosome occupancy repressed genes or the TATA less genes in NCR sensitive genes.

Earlier studies of transcriptomics and proteomics analysis of NCR metabolism have been reported^14^,^15^,^33^. This work presented here described the response of genome-wide dynamic nucleosome remodeling events to different nitrogen conditions. A correlation between NCR-sensitive genes and nucleosome profiles in response to different nitrogen condition changes was achieved. The expression of NCR-sensitive genes involving transcription regulation showed greater correlation with nucleosome prediction of nucleosome occupancy induced genes or the TATA containing genes in NCR sensitive genes. Nucleosome information involving histone modification and various types of gene regulation might result in the global regulation of gene expression^27^,^31^. Furthermore, it raises the possibility the gene patterns classified by nucleosome profiles and the assignment of transcriptional regulation factors binding at promoters might be another sketch of the overall transcription model.

## Materials and Methods

### Strains, media and culture conditions

*S. cerevisiae* strain S288C was used in this study^34^. Yeast was grown on yeast nitrogen base (YNB) medium without ammonia, without amino acids and with 3% (w/v) glucose, supplemented with 1 g/L glutamine (M.Gln) or 1 g/L proline (M.Pro) as the sole nitrogen source. The shift from M.Gln to M.Pro (M.Gln-Pro) was achieved by recovering then filtering the cells grown on M.Gln and cultivating them in fresh M.Pro for 2 h^14^. YPD medium: 10 g/L yeast extract, 20 g/L peptone and 20 g/L glucose.

### Overview of the major data

We used a MNase-seq method to isolate mononucleosome-associated DNA from *S. cerevisiae* S288C grown in media with different sole sources of nitrogen (M.Gln, M.Pro and M.Gln-Pro) and then the ends of the DNA fragments were sequenced. After aligning sequence reads to the reference genome, the genome positions of individual nucleosomes in the genome were estimated. The precise positions calculated by analysis corresponded to the known positions of these nucleosomes and NUCwave software was used for the mapped sequence reads^35^,^36^. Nucleosomes overlapping the TSS of gene-coding regions were defined as +1 nucleosomes and a nucleosome immediately before a +1 nucleosome was defined as a −1 nucleosome. The space between them was defined as a nucleosome-free region (NFR).

### Isolation of nucleosomal DNA

Yeast cultures (400 mL of strain S288C) were grown in YNB medium in 2 L flasks to optical density at 600 nm (OD_600_) of 0.6–0.8. Yeast cells were treated with 2% (v/v) formaldehyde for 30 min (with vigorous shaking every 10 min) and stopped with 125 mM glycine. Cells were collected, washed twice with sterile water and then suspended in 40 mL of buffer Z (1 M sorbitol, 50 mM Tris (pH 7.4), 10 mM β-mercaptoethanol) with Zymolyase^®^-20T (SEIKAGAKU BIOBUSINESS CORPORATION, Tokyo, Japan) at 0.25 mg/mL. Cells were spheroplasted at 28–30 °C for about 40 min, depending on how much time was necessary to remove the cell wall of each species completely. Spheroplasts were pelleted by centrifugation (3000*g* for 10 min) then suspended in 3.5 mL of NP buffer (1 M sorbitol, 50 mM NaCl, 10 mM Tris (pH 7.4), 5 mM MgCl_2_, 1 mM CaCl_2_ and 0.075% nonylphenoxypolyethoxylethanol (NP-40), with 1 mM β-mercaptoethanol and 500 μM spermidine freshly added). Samples (600 μL) of spheroplasts were added to six Eppendorf tubes containing a range of concentrations (typically 5–250 units per tube) of micrococcal nuclease (MNase) and incubated at 37 °C for 20 min. Reactions were stopped by the addition of 150 μL of stop buffer (5% (w/v) SDS, 50 mM EDTA), 5 μL of proteinase K (20 mg/mL) was added and reactions were incubated at 65 °C overnight. Samples were extracted with phenol/chloroform/isoamyl alcohol (25:24:1 by vol.), precipitated, with ethanol and treated with DNase-free RNase. Samples were examined by gel electrophoresis with ~90% mononucleosomal DNA, and mononucleosomal DNA was further purified by gel isolation and used for high-throughput sequencing with Illumina HiSeq 2500 (Illumina, San Diego, CA, USA)^27^.

### MNase-seq data and analysis

MNase-seq data were aligned to S288C *Saccharomyces cerevisiae* Genome Assembly. The raw MNase-seq data and processed data files were deposited at the NCBI Gene Expression Omnibus (GEO) (http://www.ncbi.nlm.nih.gov/geo) under accession numbers GSE75705.

The genome wide nucleosome occupancy maps from sequence reads under different nitrogen conditions were generated, annotated and normalized by using a bioinformatics tool, NUCwave^35^,^36^. The complete datasets could be found at the GEO with accession numbers GSE75705. The occupancy of a nucleosome was calculated as the logarithm of the estimated number of MNase-seq reads at the peak of a predicted nucleosome position. Furthermore, the average nucleosome occupancy score of each base pair over a range from −1000 bp to +1000 bp relative to TSS and the stop codon site were measured (Supplementary Table S1). The TATA-containing genes and TATA-less genes were classified according to previous description^28^. The average nucleosome occupancy score of TATA-containing genes and TATA-less genes at each base pair over a range from −1000 bp to +1000 bp relative to TSS were also measured (Supplementary Table S2). The nucleosome free region (NFR) of a gene was defined as the distance between the 5′ -coordinate of the +1 and the 3′ -coordinate of the −1 nucleosome. To further investigate nucleosome occupancy at each gene, their nucleosome occupancy levels were measured by calculating sequencing reads in NFR regions and gene coding regions (Supplementary Table S3). Gln_NFR, Pro_NFR and Gln-Pro are defined as the normalized reads at NFR of each gene; Gln_gene, Pro_gene and Gln-Pro_gene are defined as the normalized reads at gene coding regions of each gene. By comparing the normalized reads in the NFRs of each gene under different nitrogen conditions, we defined the values of Gln_NFR/Pro_NFR or Gln_NFR/Gln-Pro_NFR as the activation (>1) and repression (<1). A scaled difference chi-square test statistic was used to analysis the *p*-value between Gln_NFR/ Pro_NFR and Gln_NFR/Gln-Pro_NFR. There is an option to generate the statistically significant genes, which calculates an adjusted *p*-value using the Benjamini-Hochberg method, the adjusted *p*-value is *q*-value. While for TATA genes, TATA 0 represent TATA less genes; TATA 1 represent TATA containing genes.

### RNA preparation and DNA synthesis

*S. cerevisiae* S288C was grown overnight at 30 °C in YPD medium. The cells were recovered, washed and diluted to OD_600_ = 0.1 then transferred to and cultured in M.Gln until OD_600_ = 0.6–0.8. Cells were pelleted by centrifugation, washed and transferred to M.Pro for 2 h, pelleted again, washed and stored at −80 °C. The procedures for RNA extraction and cDNA synthesis are described fully elsewhere^37^.

### Quantitative real-time PCR (qRT-PCR)

Primers used for qRT-PCR were designed by Beacon Designer 7.0 software (Table 1). The qRT-PCR experiments used a SYBR^®^ Premix Ex *Taq*^TM^ kit (TaKaRa, Dalian, China). The PCR protocol was: incubation at 95 °C for 30 s then 40 cycles of amplification at 95 °C for 5 s, 55 °C for 20 s and, finally, cooling at 50 °C for 30 s. Reactions (in triplicate) took place in a LightCycler 480 II Real-time PCR instrument (Roche Applied Science, Mannheim, Germany) and mean values were used for further calculations. The fold change was determined by the 2^–ΔΔ*C*T^ method normalized to the *ACT1* gene^38^.

## Additional Information

**How to cite this article**: Zhang, P. *et al*. Genome-wide mapping of nucleosome positions in *Saccharomyces cerevisiae* in response to different nitrogen conditions. *Sci. Rep.* **6**, 33970; doi: 10.1038/srep33970 (2016).

## Supplementary Material

Supplementary Information

Supplementary Dataset 1

Supplementary Dataset 2

Supplementary Dataset 3

Supplementary Dataset 4

## Figures and Tables

**Table 1:** Oligonucleotides used for qRT-PCR.

Gene	Name	Sequence (5′-3′)
*YGK3*	*YGK3*-F	TGTACGTCAGGGAAGGGAAA
	*YGK3*-R	GCATAAGGTCCTAGCCATTCAAT
*DUR1,2*	*DUR1,2*-F	ATGTATCTGGTGGTTCCT
	*DUR1,2*-R	GCCAATCAGGTTGTTCAA
*AVT4*	*AVT4*-F	GCTGGAGGCGACATAACG
	*AVT4*-R	AACCACTTGCTGATGACAATTTAG
*ARG1*	*ARG1*-F	TTCTTACGAGGCAGGTATCTTG
	*ARG1*-R	AAGTCTTGTTGTCGGTGTAGG
*CAR1*	*CAR1*-F	ACGGAATTAGAGCCCTCAATG
	*CAR1*-R	GGAATCTGTTCGCCTGGAC
*DAL1*	*DAL1*-F	AGACGGAGCCACCTACTT
	*DAL1*-R	CACTACTGAGCCTATAACACCTTC
*GAP1*	*GAP1*-F	GAAGCACCACTTGAAGAATAG
	*GAP1*-R	CCAGAGCCATAACCATAGC
*CAN1*	*CAN1*-F	ATGGAGGATGGCATAGGTGATGA
	*CAN1*-R	GCGTTGGTCAGAGGTGTGGATA
*BAT2*	*BAT2*-F	TCGTCCAGATATGAATATG
	*BAT2*-R	CAGGAACTAAGCACTTAT
*YJR011C*	*YJR011C-F*	CGCTTATTATTGGATAGTTGTGAGT
	*YJR011C-R*	CGACATTTAATCTGTTTAATTCCTGG
*PEP4*	*PEP4*-F	CCATACGATTACACGCTTGAAGT
	*PEP4*-R	CGCAAGAAGGCATCACCAA
*UGA4*	*UGA4*-F	ATCTGTGATGGGTGGTGG
	*UGA4*-R	AGCGGTAGGAATGGAACTTG
*TMC1*	*TMC1*-F	CCACGCTAAGATAAAGAAATCAGA
	*TMC1*-R	CCGCACTTGAACAGGTATC
*RTG3*	*RTG3*-F	GAGGAAGCAGAATCGCAATCG
	*RTG3*-R	ACAGGTGAGTCGTAAGCCATAG
*PRB1*	*PRB1*-F	AAGGTGGAGGAGAAGAAGATG
	*PRB1*-R	CAGTGGACACGAGAGGAG
*APG14*	*APG14*-F	GGTCCGTCTCGCTATATTGG
	*APG14*-R	ATGAGGTCCTGTGACTGTTG
*DAL80*	*DAL80*-F	GTGCTTAGTGATTCGTTGA
	*DAL80*-R	ACCGTGTTCATCTCTTCT
*MIG2*	*MIG2*-F	CAGGCAGTAAATGGAGGTA
	*MIG2*-R	AATTGGCATAGGAGAAGTTG
*MOH1*	*MOH1*-F	TGAGCTTCATCACCTACGGTTGTA
	*MOH1*-R	GTCGCCAGTCAACATTCTTCGT
*ECM37*	*ECM37*-F	GCCACCTGATTCCTCGTCAA
	*ECM37*-R	GCGAATGCCCTGTCTTACTCA
*YLR257W*	*YLR257W*-F	CTCCTTCTCTGCGTGCTTCCA
	*YLR257W*-R	CGTCGTCTGCCTCACCTGTAAT
*GDH2*	*GDH2*-F	CCATGCTCGTGTGAGGAACT
	*GDH2*-R	CGAACACCCAGAGAAGTCATACC
*MEP3*	*MEP3*-F	GGTGGTTGGTTGACGCATA
	*MEP3*-R	TCGGCTTCCTCAGTGACT
*URK1*	*URK1*-F	AGGTATAGGTGGTGCTTCAGGTT
	*URK1*-R	GGCTCTGGCTCTGTCCTCTG
*GLN1*	*GLN1*-F	CGCCAAGGAAGGTTACGGTTA
	*GLN1*-R	CTTCGTCATGTCAGCATTGTCAA
*DIP5*	*DIP5*-F	TCATCCACCTCTACATCACCTTCA
	*DIP5*-R	GAACCACCAATGGCGATCATAGAA
*PUT4*	*PUT4*-F	CTGAGATTCCGCAAGGCTATT
	*PUT4*-R	CCCGTTGGTAATGGTGATGAT
*YHM1*	*YHM1*-F	TGCCTCATACCGATAAGAAACAAT
	*YHM1*-R	TTGGTGTGGTTGGACATCAG
*GZF3*	*GZF3*-F	GCGAAGAAGAAGGGCATATTGG
	*GZF3*-R	GCTCCTGCTTGCGATTCAC
*VID30*	*VID30*-F	TGACGACGACGACGATGA
	*VID30*-R	TTCCTTCTTCTCCACCTTCCT
*DAL5*	*DAL5*-F	GGAAGACAGAGGAACAATTCACAA
	*DAL5*-R	GGTTCTCCAGCCTTTAATAGCATA
*DAL4*	*DAL4*-F	GGAGACCACTTACACCAGAG
	*DAL4*-R	CCGTCATATCAGCACCACAT
*DAL7*	*DAL7*-F	GGAGTACAGGTTGAAGGATGACAT
	*DAL7*-R	GAAGATGGAAGCACTAATCGGTTC
*MEP2*	*MEP2*-F	AAGTGGACTACAGTTGGT
	*MEP2*-R	AACAGCAAGGTTACATCC
*CPS1*	*CPS1*-F	TTATTATCATCGGCACCTTCTTC
	*CPS1*-R	GGACTTAATGGTTCAATCTTCTCA
*NIT1*	*NIT1*-F	TGAGAAGGAGATCAAGGAATC
	*NIT1*-R	TTCGGCAAGATACTTAGCATA
*GAT1*	*GAT1*-F	GACGATGACGATGATGATGAC
	*GAT1*-R	ACCAGAAGCATTATGTGAAGC
*DSD1*	*DSD1*-F	GAATGGTATGTAGCAAGGGTCTCT
	*DSD1*-R	ACAAGCGTGTTGAGGAAGGA
*ALD4*	*ALD4*-F	GAAGGTAGAGAGGACGATGT
	*ALD4*-R	ACCGTTCCAAGACCCATTA
*PUT2*	*PUT2*-F	AGAGTCAGCGGATGGTACTTGGAA
	*PUT2*-R	TAATTTGAAAGGGCAGCGGTTTGT
*UGA1*	*UGA1*-F	GCCGTACTATCGTTCAAGAGA
	*UGA1*-R	GCCAAGCAATGGTCATCCT
*ARG4*	*ARG4*-F	CGTCTCTTCCGTATGATTA
	*ARG4*-R	CCAATTCTGTCTCCGTTA
*ACT1*	*ACT1*-F	GGTTGCTGCTTTGGTTATTG
	*ACT1*-R	CCTTGGTGTCTTGGTCTAC

## References

[b1] Kornberg R. D. Chromatin structure: a repeating unit of histones and DNA. Science 184, 868–871 (1974). 4825889 10.1126/science.184.4139.868

[b2] Bednar J. . Nucleosomes, linker DNA, and linker histone form a unique structural motif that directs the higher-order folding and compaction of chromatin. Proc. Natl. Acad. Sci. USA 95, 14173–14178 (1998). 9826673 10.1073/pnas.95.24.14173 PMC24346

[b3] Ngo T. T. & Ha T. Nucleosomes undergo slow spontaneous gaping. Nucleic. Acids Res. 43, 3964–3971 (2015). 25824950 10.1093/nar/gkv276 PMC4417179

[b4] Mobius W., Osberg B., Tsankov A. M., Rando O. J. & Gerland U. Toward a unified physical model of nucleosome patterns flanking transcription start sites. Proc. Natl. Acad. Sci. USA 110, 5719–5724 (2013). 23509245 10.1073/pnas.1214048110 PMC3619296

[b5] McKittrick E., Gafken P. R., Ahmad K. & Henikoff S. Histone H3.3 is enriched in covalent modifications associated with active chromatin. Proc. Natl. Acad. Sci. USA 101, 1525–1530 (2004). 14732680 10.1073/pnas.0308092100 PMC341768

[b6] Zaugg J. B. & Luscombe N. M. A genomic model of condition-specific nucleosome behavior explains transcriptional activity in yeast. Genome Res. 22, 84–94 (2012). 21930892 10.1101/gr.124099.111 PMC3246209

[b7] Soriano I., Morafraile E. C., Vazquez E., Antequera F. & Segurado M. Different nucleosomal architectures at early and late replicating origins in *Saccharomyces cerevisiae*. BMC Genomics 15 (2014). 10.1186/1471-2164-15-791 PMC4176565 25218085

[b8] Jiang C. & Pugh B. F. Nucleosome positioning and gene regulation: advances through genomics. Nat. Rev. Genet. 10, 161–172 (2009). 19204718 10.1038/nrg2522 PMC4860946

[b9] Zaman S., Lippman S. I., Zhao X. & Broach J. R. How *Saccharomyces* responds to nutrients. Annu. Rev. Genet. 42, 27–81 (2008). 18303986 10.1146/annurev.genet.41.110306.130206

[b10] Beltran G., Novo M., Rozes N., Mas A. & Guillamon J. M. Nitrogen catabolite repression in *Saccharomyces cerevisiae* during wine fermentations. FEMS Yeast Res. 4, 625–632 (2004). 15040951 10.1016/j.femsyr.2003.12.004

[b11] Hofman-Bang J. Nitrogen catabolite repression in *Saccharomyces cerevisiae*. Mol. Biotechnol. 12, 35–73 (1999). 10554772 10.1385/MB:12:1:35

[b12] Ljungdahl P. O. & Daignan-Fornier B. Regulation of amino acid, nucleotide, and phosphate metabolism in *Saccharomyces cerevisiae*. Genetics 190, 885–929 (2012). 22419079 10.1534/genetics.111.133306 PMC3296254

[b13] Magasanik B. & Kaiser C. A. Nitrogen regulation in *Saccharomyces cerevisiae*. Gene 290, 1–18 (2002). 12062797 10.1016/s0378-1119(02)00558-9

[b14] Scherens B., Feller A., Vierendeels F., Messenguy F. & Dubois E. Identification of direct and indirect targets of the Gln3 and Gat1 activators by transcriptional profiling in response to nitrogen availability in the short and long term. FEMS Yeast Res. 6, 777–791 (2006). 16879428 10.1111/j.1567-1364.2006.00060.x

[b15] Godard P. . Effect of 21 different nitrogen sources on global gene expression in the yeast *Saccharomyces cerevisiae*. Mol. Cell Biol. 27, 3065–3086 (2007). 17308034 10.1128/MCB.01084-06 PMC1899933

[b16] Field Y. . Distinct modes of regulation by chromatin encoded through nucleosome positioning signals. PLoS Comput. Biol. 4, e1000216 (2008). 18989395 10.1371/journal.pcbi.1000216 PMC2570626

[b17] Wei G., Hu G. Q., Cui K. R. & Zhao K. J. Genome-wide mapping of nucleosome occupancy, histone modifications, and gene expression using next-generation sequencing technology. Nucleosomes, Histones & Chromatin, Pt B 513, 297–313 (2012). 10.1016/B978-0-12-391938-0.00013-6 22929775

[b18] Cole H. A., Howard B. H. & Clark D. J. Genome-wide mapping of nucleosomes in yeast using paired-end sequencing. Nucleosomes, Histones & Chromatin, Pt B 513, 145–168 (2012). 10.1016/B978-0-12-391938-0.00006-9 22929768

[b19] Clark D. J. Nucleosome positioning, nucleosome spacing and the nucleosome code. J. Biomol. Struct. Dyn. 27, 781–793 (2010). 20232933 10.1080/073911010010524945 PMC2935628

[b20] Cui F., Chen L., LoVerso P. R. & Zhurkin V. B. Prediction of nucleosome rotational positioning in yeast and human genomes based on sequence-dependent DNA anisotropy. BMC Bioinformatics 15, 313 (2014). 25244936 10.1186/1471-2105-15-313 PMC4261538

[b21] Zhang Z. . A packing mechanism for nucleosome organization reconstituted across a eukaryotic genome. Science 332, 977–980 (2011). 21596991 10.1126/science.1200508 PMC4852979

[b22] Lee W. . A high-resolution atlas of nucleosome occupancy in yeast. Nat. Genet. 39, 1235–1244 (2007). 17873876 10.1038/ng2117

[b23] Mavrich T. N. . A barrier nucleosome model for statistical positioning of nucleosomes throughout the yeast genome. Genome Res. 18, 1073–1083 (2008). 18550805 10.1101/gr.078261.108 PMC2493396

[b24] Hoffman B. G. . Locus co-occupancy, nucleosome positioning, and H3K4me1 regulate the functionality of FOXA2-, HNF4A-, and PDX1-bound loci in islets and liver. Genome Res. 20, 1037–1051 (2010). 20551221 10.1101/gr.104356.109 PMC2909568

[b25] Yang L. . Characterization of TATA-containing genes and TATA-less genes in *S. cerevisiae* by network topologies and biological properties. Genomics 104, 562–571 (2014). 25451177 10.1016/j.ygeno.2014.10.005

[b26] Huang H., Chen J., Liu H. & Sun X. The nucleosome regulates the usage of polyadenylation sites in the human genome. BMC Genomics 14, 912 (2013). 24365105 10.1186/1471-2164-14-912 PMC3879661

[b27] Shivaswamy S. . Dynamic remodeling of individual nucleosomes across a eukaryotic genome in response to transcriptional perturbation. PLoS. Biol. 6, 618–630 (2008). 10.1371/journal.pbio.0060065 PMC2267817 18351804

[b28] Basehoar A. D., Zanton S. J. & Pugh B. F. Identification and distinct regulation of yeast TATA box-containing genes. Cell 116, 699–709 (2004). 15006352 10.1016/s0092-8674(04)00205-3

[b29] Zawadzki K. A., Morozov A. V. & Broach J. R. Chromatin-dependent transcription factor accessibility rather than nucleosome remodeling predominates during global transcriptional restructuring in *Saccharomyces cerevisiae*. Mol Biol Cell 20, 3503–3513 (2009). 19494041 10.1091/mbc.E09-02-0111 PMC2719568

[b30] Kristell C. . Nitrogen depletion in the fission yeast *Schizosaccharomyces pombe* causes nucleosome loss in both promoters and coding regions of activated genes. Genome Res. 20, 361–371 (2010). 20086243 10.1101/gr.098558.109 PMC2840984

[b31] Workman J. L. & Kingston R. E. Alteration of nucleosome structure as a mechanism of transcriptional regulation. Annu. Rev. Biochem. 67, 545–579 (1998). 9759497 10.1146/annurev.biochem.67.1.545

[b32] Erb I. & van Nimwegen E. Transcription factor binding site positioning in yeast: proximal promoter motifs characterize TATA-less promoters. PloS One 6, e24279 (2011). 21931670 10.1371/journal.pone.0024279 PMC3170328

[b33] Zhao S. . Comparative proteomic analysis of *Saccharomyces cerevisiae* under different nitrogen sources. J. Proteomics 101, 102–112 (2014). 24530623 10.1016/j.jprot.2014.01.031

[b34] Brachmann C. B. . Designer deletion strains derived from *Saccharomyces cerevisiae* S288C: a useful set of strains and plasmids for PCR-mediated gene disruption and other applications. Yeast 14, 115–132 (1998). 9483801 10.1002/(SICI)1097-0061(19980130)14:2<115::AID-YEA204>3.0.CO;2-2

[b35] Quintales L., Vazquez E. & Antequera F. Comparative analysis of methods for genome-wide nucleosome cartography. Brief Bioinform. 16, 576–587 (2015). 25296770 10.1093/bib/bbu037

[b36] Tsui K. . Evolution of nucleosome occupancy: conservation of global properties and divergence of gene-specific patterns. Mol. Cell Biol. 31, 4348–4355 (2011). 21896781 10.1128/MCB.05276-11 PMC3209338

[b37] Zhao X. R. . Metabolic engineering of the regulators in nitrogen catabolite repression to reduce the production of ethyl carbamate in a model rice wine system. Appl. Environ. Microbiol. 80, 392–398 (2014). 24185848 10.1128/AEM.03055-13 PMC3910993

[b38] Boer V. M. . Transcriptional responses of *Saccharomyces cerevisiae* to preferred and nonpreferred nitrogen sources in glucose-limited chemostat cultures. FEMS Yeast Res. 7, 604–620 (2007). 17419774 10.1111/j.1567-1364.2007.00220.x

