# Supplementary material for: Genome-wide mapping of nucleosome positions in Saccharomyces cerevisiae in response to different nitrogen conditions
Source: Sci Rep. 2016 Sep 23;6:33970. doi: 10.1038/srep33970 (PMC5034280; doi:10.1038/srep33970)
Supplement: Supplementary Information [file srep33970-s1.pdf]

Genome-wide mapping of nucleosome positions in *Saccharomyces cerevisiae* in response to different nitrogen conditions

Peng Zhang[1], Guocheng Du[1], Huijun Zou[2], Guangfa Xie[2], Jian Chen[1], Zhongping Shi[1*], Jingwen Zhou[1*]

[1] Key Laboratory of Industrial Biotechnology, Ministry of Education and School of Biotechnology, Jiangnan University, 1800 Lihu Road, Wuxi, Jiangsu 214122, China;

[2] Zhejiang Guyuelongshan Shaoxing Wine Company, 13 Yangjiang Road, Shaoxing, Zhejiang, China;

[*] Corresponding authors.

Zhongping Shi, Jingwen Zhou

Mailing address: School of Biotechnology, Jiangnan University, 1800 Lihu Road, Wuxi, Jiangsu 214122, China.

Phone: +86-510-85918312, Fax: +86-510-85918309

E-mail: zpshi@jiangnan.edu.cn, zhoujw1982@jiangnan.edu.cn.

Supplementary information

Supplementary Table S1 Average nucleosome occupancy score of each base pair over TSS and the stop codon site in response to different nitrogen conditions

Supplementary Table S2 Average nucleosome occupancy score of TATA containing and TATA less genes in response to different nitrogen conditions

Supplementary Table S3 Nucleosome occupancy levels at NFRs and gene coding regions in response to different nitrogen conditions.

The nucleosome data were mapped and annotated to S288C genome, sequencing reads in NFR regions and gene coding regions in different nitrogen conditions

Supplementary Table S4 qRT-PCR analysis of the nucleosome occupancy changed NCR sensitive genes in different nitrogen conditions.

More than 80 NCR sensitive genes were listed, 24 (M.Pro vs M.Gln) and 26 (M.Gln-Pro vs M.Gln) genes were analyzed by qRT-PCR, respectively.
